# Maternal use of prednisolone is unlikely to be associated with neonatal adrenal suppression—a single-center study of 16 cases

**DOI:** 10.1007/s00431-017-2949-1

**Published:** 2017-07-10

**Authors:** Leanne de Vetten, Margriet van Stuijvenberg, Ido P. Kema, Gianni Bocca

**Affiliations:** 1 Department of Pediatrics, University Medical Center Groningen, Groningen, Netherlands; 2 Beatrix Children’s Hospital, University Medical Center Groningen, P.O. 30.001, 9700 RB Groningen, Netherlands; 3 Department of Laboratory Medicine, University Medical Center Groningen, Groningen, Netherlands

**Keywords:** Adrenal function, Adrenal suppression, Hypothalamic-pituitary-adrenal axis, Neonatal endocrinology, Prednisolone

## Abstract

The use of supra-physiological, exogenous corticosteroids in pregnancy may lead to neonatal adrenal suppression. We report on a single-center, case series study carried out between 2006 and 2014, which included all newborns (*n* = 16) of mothers using prednisolone ≥10 mg/day during pregnancy. Newborns were routinely assessed according to hospital protocol, with follow-up until 6 weeks after birth. We investigated the clinical symptoms and biochemical findings of adrenal suppression occurring in the newborns. Mean dose of maternal prednisolone was 29.7 ± 16.1 mg/day with a mean duration of 18.4 ± 15.4 weeks. Five newborns showed hypoglycemia with normal serum cortisol concentrations and urinary steroid profiles. Two newborns had abnormal urinary steroid profiles, probably the result of prematurity, but with adequate adrenal stress response during clinical sepsis.

*Conclusion*: In this retrospective case series, we found no evidence of prolonged effects of maternal prednisolone use during pregnancy on the *neonatal* hypothalamic-pituitary-adrenal axis.

**Table:** 

**What is known:** • *The use of prednisolone during pregnancy may cause increased steroid levels in the fetus by partially passing through the placenta.* • *So far, there was very limited data available on the occurrence of adrenal suppression in the newborn of mothers using prednisolone during pregnancy.*	
**What is new:** • *The use of high-dosage prednisolone during pregnancy for ≥1 week (mean duration of 18.4 ± 15.4 weeks), prior to delivery, appears to have little influence on the neonatal hypothalamic-pituitary-adrenal axis.*

## Introduction

Reproduction in women with serious medical problems, such as chronic inflammatory bowel disease or asthma, has become increasingly common. The associated use of medication, including systemic corticosteroids, in pregnant women may incur an increased risk for the fetus or newborn baby.

Prednisolone is the drug of choice as the estimated fetal level is only 10% of the maternal level [6]. This is the result of the conversion of prednisolone to less active metabolites by the enzyme 11β-hydroxysteroid dehydrogenase-2 (11-beta-HSD2) in the placenta [6]. There is less wide experience with the use of hydrocortisone during pregnancy. Dexamethasone is only partially metabolized by the enzyme 11-beta-HSD2, and up to 50% reaches the fetus [6]. When taken in high doses and over a longer period of time, prednisolone can saturate the placental enzymes, after which, larger amounts of corticosteroids will cross the placental barrier [1].

Chronic use of corticosteroids may result in suppressive effects on the *neonatal* hypothalamic-pituitary-adrenal (HPA) axis [8]. Then, in times of physical stress, such as an infection, the adrenal gland may not be able to produce sufficient hormone (cortisol) to help the newborn to respond adequately. The lack of this response (Addisonian crisis) is a life-threatening situation for which supplementation of corticosteroids is needed.

So far, there was no clinical data on the risk of HPA axis suppression in newborns whose mothers had been using systemic corticosteroids for a longer period during pregnancy. Neither were there any studies on the influence of dosage and duration of maternal prednisolone use during pregnancy. Local guidelines for monitoring HPA axis suppression in newborns were therefore developed in the UMCG, covering admission to the pediatric ward or neonatal intensive care unit (NICU), assessment of blood pressure, blood glucose levels, serum cortisol and plasma adrenocorticotropic hormone (ACTH) levels, urinary steroid profile (USP), and, if necessary, dynamic testing of the HPA axis. These measures lead to a distressing separation of mother and child and pose a substantial financial burden on the healthcare budget. Based on the current pathophysiological knowledge, we would expect a substantial number of cases to show HPA axis suppression. However, our clinical experience is that its prevalence, and that of refractory hypoglycemia and hypotension, is very low.

Our aim was to determine the clinical symptoms and biochemical signs of HPA axis suppression occurring in newborns whose mothers had used systemic prednisolone during their pregnancy.

## Methods

### Patient population

We included all live-born infants whose mothers had used ≥10 mg/day of prednisolone (orally or intravenously) during ≥1 week prior to delivery, born in an 8-year period between October 2006 and October 2014 in the University Medical Center Groningen (UMCG). The UMCG is a tertiary hospital with about 1600 deliveries per year, and it is the only hospital in the region with a NICU. In the region covered by our hospital, all obstetricians and midwives must consult a neonatologist and pediatric endocrinologist if they treat a pregnant woman using systemic corticosteroids for a longer period of time and/or prior to delivery.

### Data collection

Maternal and pediatric data were collected retrospectively from medical files.

### Guideline procedures

The neonatologist is consulted for every live-born infant of mothers who used prednisolone prior to delivery. For mothers who used ≥10 mg/day of prednisolone, their newborn was admitted to the maternity ward. The child was admitted to the NICU for 48 h observation if the mother used ≥35 mg/day of prednisolone. All newborns were routinely assessed by physical examination and laboratory tests during their first 48 h. Dynamic testing of the HPA axis was considered only in cases of persistent findings of adrenal suppression. Newborns were followed up until 6 weeks after birth.

### Definition of outcome measures

*Blood pressure* was measured (by full-automatic oscillometric measurement device), during the first 48 h of life and, after 48 h, when clinically indicated. Hypotension was defined as a mean blood pressure in mmHg below the tenth percentile for gestational and postnatal age.

*Serum glucose level* was measured by an enzymatic method (hexokinase-mediated reaction, Roche Modular, Mannheim, Germany) and assessed in all newborns, every 3 h for their first 48 h. Samples were taken before oral feedings. Hypoglycemia was defined as a blood glucose concentration <2.6 mmol/L (46 mg/dL).

*Serum cortisol and plasma ACTH level* was assessed in all newborns within the first 48 h. Ethylenediaminetetraacetic acid **(**EDTA) tubes were used to collect blood samples for ACTH, which were placed on ice and, after centrifugation, frozen immediately until analysis. The analysis was performed by electrochemiluminescence immunoassay using Cobas 6000 E601 (Roche Diagnostics, intra-assay coefficient of variation (CV) 0.6–3.6% and inter-assay CV 3.5–5.4%). Plasma cortisol was analyzed by electrochemiluminescence immunoassay using Modular E170 (Roche Diagnostics, inter-assay CV 2.3–4.0%). As newborns do not have a circadian rhythm of cortisol secretion, samples were taken at random moments during the day [4]. In screening adrenal dysfunction, a combined measurement of ACTH and cortisol in blood is used as standard practice at the UMCG. Adrenal dysfunction was defined if the ACTH level was >45.5 ng/L (10 pmol/L) and/or the cortisol level was <200 nmol/L in full-term newborns or 100 nmol/L in pre-term newborns. These cutoff levels have been used for many years as institutional reference ranges in clinical practice at the UMCG and are in accordance with the literature [7, 9].

*Urinary steroid profile* (USP) was assessed in all newborns in the first 48 h by taking a sample from a urine collection bag [2]. After enzymatic hydrolysis of urine samples, steroids were extracted and derivatized. Derivatized samples were analyzed using quantitative gas chromatography/mass spectrometry (GC/MS) with capillary chromatography. If fetal metabolites were absent, the USP was defined as abnormal and repeated after 2–4 weeks.

## Results

### Patient population

We enrolled 16 newborns in the study. Newborn and maternal characteristics are described in Table 1. Gestational age varied from 26 2/7 to 41 3/7 weeks (mean 34 5/7 ± 4 4/7 weeks) and birth weight varied from 885 to 4175 g (mean 2477 ± 1002 g). The dose and duration of prednisolone use varied from 10 to 60 mg/day (mean 29.7 ± 16.1 mg/day) and from one to 40 weeks (mean 18.4 ± 15.4 weeks) prior to delivery.

**Table 1:** Maternal and newborn characteristics of 16 cases, ranked by mother’s cumulative dose of prednisolone during pregnancy

	Cumulative dose of prednisolone	Maternal age	Maternal medical history	Dose	Duration of treatment	Complications of pregnancy	Birth	Newborn sex	Gestational age	Birth weight	Hypoglycemia	Hypo-tension	Urinary steroid profile	Serum cortisol	Plasma ACTH
	(mg)	(years)		(mg/ day)	(weeks)				(weeks)	(g)	(mmol/L)	mean (mmHg)		(nmol/L)	(ng/L)
1	210	25	Asthma	10	3	None	SVD	M	41 3/7	4120	No	–	Normal	–	–
2	210	23	Asthma, obesity	30	1	Diabetes gravidarum	Secondary C/S	M	37 4/7	3715	Yes; 1.8	–	Normal	2660	–
3	245	32	PUPPP	35	1	None	Emergency C/S	F	38	4175	Yes; 1.5	No	Normal	–	–
4	315	18	Pemphigus gravidarum	15	3	None	SVD	M	35 5/7	2285	Yes; 1.6	–	Normal	335	38
5	630	26	Pemphigus gravidarum, hypothyroidism, DVT	30	3	Diabetes gravidarum	SVD	M	26 2/7	1155	No	–	Normal	635	–
6	1400	22	Cystic fibrosis	50	4	PROM	Secondary C/S	M	28 1/7	1135	No	No	Normal	450	–
7	2240	19	Auto-immune hemolytic anemia	40	8	None	SVD	F	35 4/7	2685	No	No	Normal	230	-
8	2310	37	Auto-immune hemolytic anemia	10	33	Diabetes gravidarum	Emergency C/S	F	33 2/7	1920	Yes; <1.1	–	Normal	650	7.7
9	2450	33	Mixed connective tissue disease	12.5	28	PIH, IUGR	Emergency C/S	F	28	885	No	Yes; 25^a^	Abnormal	670	–
10	3150	23	Tuberculous meningitis	50	9	None	SVD	F	37 4/7	2880	No	No	Normal	680	26
11	3412	32	Asthma	12.5	39	Infection	Secondary C/S	F	39 5/7	2810	No	–	Normal	80	14
12	4200	34	Systemic lupus erythematosus	15	40	Infection	SVD	F	40	3120	No	–	Normal	840	8.6
13	5460	36	Asthma	30	26	None	Secondary C/S	F	35 5/7	2690	No	–	Normal	105	39.7
14	7350	41	Colitis ulcerosa	30	35	None	SVD	F	35 4/7	2190	No	–	Normal	325	38
15	9765	35	Muscle disorder n.o.s., asthma, hypothyroidism	45	31	Pulmonary embolism	Emergency C/S	F	31 5/7	2100	Yes; 1.2	No	Normal	750	21
16	12,600	27	Myasthenia gravis	60	30	Pre-eclampsia	Primary C/S	M	30 5/7	1760	No	Yes; 27^b^	Abnormal	427	17

*PUPPP* pruritis urticarial papules and plaques of pregnancy, *DVT* deep venous thrombosis, *PIH* pregnancy-induced hypertension, *IUGR* intra-uterine growth restriction, *N.o.s* not otherwise specified, *PROM* premature rupture of membranes, *SVD* spontaneous vaginal delivery, *C/S* cesarean section

^a^culture-negative sepsis (15th day after birth)

^b^Staphylococcus Aureus sepsis (5th day after birth)—missing data

### Outcome

Five newborns had transient hypoglycemia (Table 1). They all showed well-known risk factors for hypoglycemia (prematurity (*n* = 3), birth weight large for gestational age (*n* = 1), and maternal insulin-dependent diabetes gravidarum (*n* = 1)). They received extra oral feedings and/or intravenous glucose administration according to the UMCG guideline for neonatal hypoglycemia. All five newborns had normal serum cortisol levels and USP.

One newborn (patient 11, Table 1) showed a low serum cortisol level of 80 nmol/L. She did not have hypoglycemia or hypotension. The USP showed presence of fetal metabolites. Therefore, dynamic testing of the HPA axis was not performed.

In two newborns (patients 9 and 16, Table 1), USP from a urine sample collected on the first day of life showed the absence of fetal metabolites. Both infants were born pre-term, did not show hypoglycemia, but did have hypotension during an episode of clinical sepsis (patient 9 on the 15th day and patient 16 on the 5th day after birth). They were treated with a single intravenous fluid bolus with saline and broad-spectrum antibiotics. They did not receive steroid replacement therapy. Serum cortisol levels were in the normal range. Within 4 weeks, the USP of both children contained neonatal steroid hormone metabolites.

## Discussion

In this retrospective analysis during a time period of 8 years, we found 16 live-born children of mothers using ≥10 mg/day of prednisolone for ≥1 week prior to delivery. The chronic use of high-dosed prednisolone during pregnancy is thus not of frequent occurrence. We found no clinical symptoms of adrenal suppression, only small abnormalities in the laboratory results of half of these newborns (eight out of 16) which we will describe in the following paragraphs.

Five newborns showed hypoglycemia. However, it seems unlikely that these cases were related to HPA axis suppression as all had a normal USP and normal serum cortisol levels. Furthermore, they all had known risk factors for hypoglycemia (e.g., prematurity, birth weight large for gestational age, and maternal insulin-dependent diabetes gravidarum) and would therefore have been detected through our local hospital guideline.

We found one newborn (patient 11) with a low serum cortisol level (80 nmol/L). As newborns do not have a circadian rhythm of cortisol secretion [4], this finding might have been the result of taking samples on random moments during the day. We did not find USP abnormalities or clinical symptoms suggesting HPA axis suppression in this newborn.

In two newborns (patients 9 and 16), the first USP lacked fetal metabolites although serum cortisol levels were normal. During an episode of clinical late-onset sepsis, both newborns developed hypotension, for which they received a single intravenous fluid bolus with saline and broad-spectrum antibiotics. They did not receive steroid replacement therapy. Within 4 weeks after birth, both had USPs containing neonatal steroid hormone metabolites. Their response to sepsis without needing steroid replacement therapy suggests that both these newborns had an adequate adrenal stress response despite a transient abnormal USP. We can thus conclude that both pre-terms showed transient abnormal USP without clinical symptoms of adrenal insufficiency.

Since both these newborns were pre-term (28 and 30 weeks of gestational age), the immaturity of their HPA axis might have affected the USP. Furthermore, the enzymatic activity of 11-beta-HSD2 in the placenta could have been decreased because of immaturity, resulting in a higher transplacental passage of prednisolone. Indeed, a positive association between gestational age and 11-beta-HSD2 activity was previously described [3].

In both cases, placental bed pathology was suspected (one mother had hemolysis with elevated liver enzymes and low platelets (HELLP) syndrome, and the other had pregnancy-induced hypertension (PIH) combined with intra-uterine growth restriction). No other cases of placental bed pathology were found in our database. Placental bed pathology might influence the function of 11-beta-HSD2, resulting in a higher (more than double) transplacental passage of prednisolone [5]. However, a more recent study found contrary evidence in women with HELLP syndrome [10], reporting prednisolone levels of about 10% of maternal levels in the umbilical cords, similar to the levels seen in women without HELLP syndrome [6].

Broad reference ranges used in newborn HPA testing may suggest lack of specificity. Serum cortisol can be affected by an intrinsic production failure (due to immaturity of the HPA axis), as well as extrinsic factors such as stress during and after delivery [7]. We therefore feel that the interpretation of normal serum cortisol levels should take into account the clinical circumstances and the presence or absence of clinical signs of adrenal insufficiency. Additional information on the adrenal function can be provided by USP. Dynamic function testing of the HPA axis (e.g., ACTH stimulation testing) can confirm adrenal insufficiency by measuring the adrenal response to stress, and thus provides the most relevant information for clinical practice. Because our newborns did not show persistent signs of adrenal suppression, dynamic testing of the HPA axis was not considered necessary.

### Study limitations

Although this is the first case series report on the subject, the most important limitation of our study is the relatively small number of patients and its retrospective character. It is possible that the obstetricians and midwives may not have reported a mother using prednisolone during pregnancy and that we might thereby have missed a case. Yet, all newborns in our region who would have developed symptomatic adrenal insufficiency would have been admitted to our NICU.

We found a great heterogeneity in patient characteristics and a wide variation in the dosage of prednisolone and duration of treatment. Both the small number of patients and the heterogeneity in patient characteristics in our study make it difficult to exclude a possible effect of the use of maternal prednisolone on the neonatal HPA axis. This uncertainty mainly applies to the different gestational ages of the children. It could be hypothesized that the influence of maternal prednisolone use on the neonatal HPA axis is more pronounced when the gestational age of the infant is younger. A multicenter, prospective cohort study on a larger number of patients would allow analyses of sub-groups, e.g., full-term vs. pre-term born infants, effect of dosage, and duration of prednisolone treatment and would allow risk factors to be identified using logistic regression analysis. Such a cohort study would also benefit from dynamic testing of the HPA axis in newborns showing biochemical evidence of adrenal suppression, which would allow evaluation of the adrenal response to stress.

## Conclusion

In this retrospective case series of 16 newborns, we found no evidence of prolonged effects on the *neonatal* HPA axis of mothers using prednisolone during pregnancy. Small and transient biochemical abnormalities were not associated to clinical symptoms of adrenal insufficiency. However, a prospective cohort study with a large number of newborns is needed to further elucidate the effect of maternal corticosteroid use on the neonatal HPA axis.

## Abbreviations

11-beta-HSD2: 11-beta-hydroxysteroid dehydrogenase-2
ACTH: adrenocorticotropic hormone
CV: coefficient of variation
EDTA: ethylenediaminetetraacetic acid
GC/MS: gas chromatography/mass spectrometry
HELLP: hemolysis with elevated liver enzymes and low platelets
HPA: hypothalamic-pituitary-adrenal
NICU: neonatal intensive care unit
PIH: pregnancy-induced hypertension
UMCG: University Medical Center Groningen
USP: urinary steroid profile
